# Identification and Molecular Mechanisms of the Rapid Tonicity-induced Relocalization of the Aquaporin 4 Channel*

**DOI:** 10.1074/jbc.M115.646034

**Published:** 2015-05-26

**Authors:** Philip Kitchen, Rebecca E. Day, Luke H. J. Taylor, Mootaz M. Salman, Roslyn M. Bill, Matthew T. Conner, Alex C. Conner

**Affiliations:** From the ‡ Molecular Organisation and Assembly in Cells Doctoral Training Centre, University of Warwick, Coventry CV4 7AL,; the § Biomedical Research Centre, Sheffield Hallam University, Howard Street, Sheffield S1 1WB,; the ¶ School of Life & Health Sciences and Aston Research Centre for Healthy Ageing, Aston University, Aston Triangle, Birmingham B4 7ET, and; the ‖ School of Clinical and Experimental Medicine, University of Birmingham, Edgbaston, Birmingham B15 2TT, United Kingdom

**Keywords:** aquaporin, astrocyte, homeostasis, protein translocation, water channel, hypotonicity, rapid trafficking, regulation

## Abstract

The aquaporin family of integral membrane proteins is composed of channels that mediate cellular water flow. Aquaporin 4 (AQP4) is highly expressed in the glial cells of the central nervous system and facilitates the osmotically driven pathological brain swelling associated with stroke and traumatic brain injury. Here we show that AQP4 cell surface expression can be rapidly and reversibly regulated in response to changes of tonicity in primary cortical rat astrocytes and in transfected HEK293 cells. The translocation mechanism involves PKA activation, influx of extracellular calcium, and activation of calmodulin. We identify five putative PKA phosphorylation sites and use site-directed mutagenesis to show that only phosphorylation at one of these sites, serine 276, is necessary for the translocation response. We discuss our findings in the context of the identification of new therapeutic approaches to treating brain edema.

## Teaser

**Background:** The water channel protein aquaporin 4 (AQP4) controls water permeability of the blood-brain barrier.

**Results:** Hypotonicity induces rapid relocalization of AQP4 in a calcium-, calmodulin-, and kinase-dependent manner.

**Conclusion:** AQP4 can be relocalized between the cell membrane and intracellular compartments.

**Significance:** Pharmacological modulation of AQP4 membrane localization could provide a new approach to treating brain edema.

## Introduction

The aquaporins (AQPs)^4^ form a family of small integral membrane proteins found in all phylogenetic kingdoms. They act as channels for the osmotic passage of water through biological membranes and they mediate cellular water flow.

Human AQP4 is a relatively recently discovered member of the 13-strong family of mammalian AQPs (1). AQP4 is found in a number of tissues including the kidney and gastrointestinal tract but is notable for its high levels in astrocytes and its role in brain water homeostasis (2).

AQP4 has been shown to be important for brain edema formation following traumatic brain injury, stroke, and meningitis (3). AQP4 knock-out mice were protected from the formation of cytotoxic edema in a stroke model (4), providing an explicit target for managing this condition. Despite extensive research that has built on the well understood structural biology of the AQP family, there are no drugs available to restrict water movement through AQP4, and in general, known AQP inhibitors are either cytotoxic (mercurial compounds) or nonspecific, leading to off-target effects (5–7). Understanding the regulation of AQP4 now provides a novel therapeutic approach for the prevention of cytotoxic edema.

The most well studied system in which cellular water flow is regulated is the translocation of AQP2 in response to vasopressin (Arg-8 vasopressin (AVP); anti-diuretic hormone (ADH)) in the cells of the collecting duct of the mammalian kidney (8). AQP2 is shuttled between the apical cell surface and intracellular vesicles to regulate water reabsorption. The mechanism involves AVP-mediated production of cAMP resulting in the activation of PKA and direct phosphorylation of the AQP2 C-terminal tail at serine 256 (9). Specific SNARE proteins that facilitate the exocytosis of AQP2-containing vesicles have been identified (10). Other AQPs have been shown to be translocated in response to environmental stimuli; we recently reviewed an emerging consensus on subcellular relocalization as a regulatory mechanism for the AQP family (11) following our discovery of the PKC-mediated translocation of threonine-phosphorylated AQP1 in response to changes of tonicity (12, 13).

There is a requirement for rapid, physiological regulation of AQP4, which could be achieved at the transcriptional level or by short-term subcellular relocalization. Despite this, there is no description of a mechanism of AQP4 translocation, and the effect of stimuli on AQP4 localization is contradictory. AQP4 localization is thought to be affected by numerous intracellular mechanisms including PKA (14), PKC (15), and actin reorganization (16). It is not clear whether there are common mechanisms either within the AQP family or even for AQP4 in different cell types; this indicates a general lack of mechanistic awareness of AQP regulation.

We have identified a novel regulatory pathway of endogenous AQP4 translocation in primary cortical astrocytes. This study describes this mechanism in a model HEK293 cell line and shows how tonicity-dependent changes in the environment lead to a calcium-dependent, calmodulin-dependent, and PKA-specific, reversible translocation of AQP4 to the cell surface. We also identify a known kinase site and putative PKA target site, serine 276, in the C-terminal tail of AQP4 that is necessary for the translocation response. This site has been shown to be phosphorylated *in vivo*, and we show that the mechanism requires PKA activity in primary cortical astrocytes. Our data represent a novel mechanism that could be a new avenue for therapeutic discovery in the treatment of cytotoxic brain edema.

## Experimental Procedures

### 

#### 

##### Materials

Cell-permeable inhibitors were purchased as follows: trifluoperazine and W7 calmodulin antagonists from Sigma (Poole, UK); the myristoylated PKA inhibitor Myr-PKI 14-22 amide from Merck Chemicals (Nottingham, UK); and Myr-PKC 19-27 and hypericin from Fisher Scientific (Loughborough, UK). FluoroDish^TM^ dishes were from World Precision Instruments Ltd. (Stevenage, UK). Monoclonal rabbit anti-AQP4 antibody was from Abcam (Cambridge, UK, product code ab128906), and secondary goat anti-rabbit IgG-HRP was from Santa Cruz Biotechnology. Gateway vectors and enzymes were from Invitrogen (Paisley, UK). Unless otherwise specified, all other chemicals were from Sigma or Fisher Scientific. Cell culture reagents, including calcium-free DMEM, were from Sigma. In each experiment, all inhibitors were analyzed using uninhibited cells transfected with wild-type AQP4-GFP as a positive control.

##### Cell Culture and Transfection

HEK293 cells were cultured routinely in DMEM supplemented with 10% (v/v) fetal bovine serum in humidified 5% (v/v) CO_2_ in air at 37 °C. Cells were seeded into 35-mm FluoroDish^TM^ dishes (World Precision Instruments) and transfected after 24 h using PEI. 2 μg of DNA was added to 100 μl of pre-warmed serum-free DMEM followed by 12 μl of 1 mg/ml PEI (pH 7.4). The mixture was incubated at room temperature for 10 min. 600 μl of pre-warmed growth media was added, and cell culture media were replaced with the transfection mix. 2 ml of growth media was added after 2 h. The transfection mix was replaced by 2 ml of culture medium after a further 22 h. Transfected cells were imaged 24–36 h after transfection. Rat primary cortical astrocytes were isolated and cultured as described previously (12). All cells tested negative for mycoplasma using the EZ-PCR mycoplasma test kit (Biological Industries).

##### Expression Constructs and Mutagenesis

Human AQP4 cDNA was a kind gift of Dr. Kristina Hedfalk (University of Gothenburg). This was fused with C-terminal GFP in the pDEST47 expression vector using the Gateway cloning system (Invitrogen) as described previously (12). Site-directed mutagenesis was performed using the QuikChange procedure (Stratagene).

##### Confocal Microscopy

AQP4-GFP constructs were imaged in live cells using a Zeiss LSM780 confocal microscope with a 63 × 1.3 NA water immersion objective and an incubated stage held at 37 °C. GFP was excited using the 488-nm line of an argon laser. FM4-64 was excited using the 514-nm line of an argon laser. ER-Tracker Blue-White was excited using a 405-nm diode laser. Hypotonic challenges were achieved by dilution of medium with distilled H_2_O on the microscope stage.

##### Image Analysis

Line profiles across the cell membrane and cytoplasm were extracted using ImageJ as described previously (13) in a semi-automated fashion using an in-house ImageJ macro. Line scan positions were chosen to avoid the nucleus and perinuclear region. Relative membrane expression (RME) was calculated from profiles using an in-house MATLAB code. ER-localized AQP4 affected the calculated RME by <10%. This was confirmed by recalculating the RME after subtracting the GFP signal that co-localized with ER-Tracker Blue-White. Cell cross-sectional areas were calculated in ImageJ using the particle detection tool. Transfected and non-transfected cell swelling was compared using the FM4-64 membrane dye (see Fig. 3).

##### SDS-PAGE and Western Blotting

HEK293 cells were lysed in radioimmunoprecipitation assay buffer (pH 8.0). 2 μg of total cellular protein was electrophoresed on 8% polyacrylamide gels. Blocking buffer was 20% w/v nonfat powdered milk in PBS-Tween (0.1%). Anti-AQP4 primary antibody (Abcam, ab128906) was diluted 1:10,000 in 0.1% PBS-Tween. Donkey anti-rabbit IgG-HRP (Santa Cruz Biotechnology, sc2313) was diluted 1:20,000 in 0.1% PBS-Tween.

##### Biotinylation

Primary astrocytes were plated in 6-well plates 2 days before the experiments. Cell surface amines were biotinylated using a cell-impermeable amine-reactive biotinylation reagent (EZ-Link Sulfo-NHS-SS-Biotin, Thermo Scientific). Cells were exposed to reductions in extracellular tonicity by diluting culture medium with distilled H_2_O. Cell culture media were washed out with PBS (with Ca^2+^/Mg^2+^) while avoiding completely drying the cells. Cells were incubated in 600 μl of 0.5 mg/ml biotinylation reagent in PBS diluted to the tonicity matching the cell treatment on ice for 30 min. For basal surface expression experiments, cells were washed twice in ice-cold PBS instead of hypotonic treatment. Unreacted reagent was quenched in 25 mm glycine in PBS three times for 5 min. Cells were lysed in Tris-Triton (1%) lysis buffer (pH 7.4). The lysate was centrifuged at 21,000 × *g* at 4 °C for 10 min to remove insoluble material. Biotinylated proteins were pulled out by incubation in NeutrAvidin-coated 96-well plates (Pierce) for 2 h at 4 °C. Each lysate was loaded in triplicate with the same amount of total cellular protein (measured by BCA assay) per lysate. Plates were blocked with 3% w/v BSA in PBS for 1 h at room temperature. Plates were incubated overnight at 4 °C with an AQP4 antibody (Abcam, ab128906) diluted 1:2,000 in 0.05% PBS-Tween. Plates were washed with 0.1% PBS-Tween and incubated at room temperature for 1 h with HRP-conjugated secondary antibody (Santa Cruz Biotechnology, sc-2313). Plates were washed with 0.1% PBS-Tween and incubated with *o*-phenylenediamine dihydrochloride (Sigma-Aldrich) for 30 min, wrapped in foil. Absorbance was measured at 450 nm using a BioTek Synergy HT plate reader.

##### Osmolality

Osmolalities of all treatment solutions were measured using an Osmomat 3000 freezing point depression osmometer (Gonotec).

##### Statistics

For microscopy experiments in which the same cells were imaged before and after tonicity changes, paired *t* tests were used. For multiple treatments of the same cells, analysis of variance was used followed by paired *t* tests, with *p* values subjected to Bonferroni's correction. For biotinylation experiments in which different cells were subject to different treatments, unpaired *t* tests were used following analysis of variance and *p* values subjected to Bonferroni's correction. All *p* values referred to in the text and figures are the post-correction values, rounded up to 1 significant figure. *p* < 0.05 was considered statistically significant (*).

## Results

### 

#### 

##### AQP4 Undergoes a Rapid and Reversible Subcellular Relocalization in Primary Cortical Astrocytes in Response to Changes in the Tonicity of the Extracellular Environment

**FIGURE 1.**
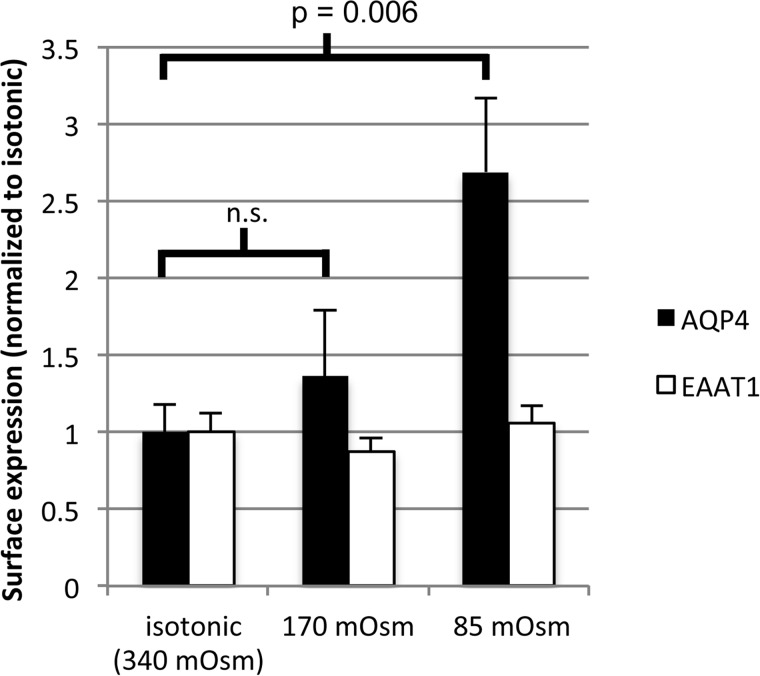
**Endogenous AQP4 relocalization in primary cortical astrocytes.** Cell surface biotinylation of primary rat astrocytes subjected to hypotonic stress and analysis of endogenous AQP4 surface expression is shown, *n* = 3. As a negative control for translocation, membrane expression of the glutamate transporter EAAT1 was measured under the same hypotonic conditions, *n* = 3. All data are presented as mean ± S.E. *p* values and significance on the graph refer to AQP4. None of the variability in EAAT1 surface expression was statistically significant, *p* = 0.37. *n.s.*, not significant.

Surface expression of endogenous AQP4 in rat primary cortical astrocytes was measured using cell surface biotinylation followed by a NeutrAvidin-based ELISA. Fig. 1 shows that AQP4 surface localization increased 2.7-fold after 10 min of hypotonic challenge at 85 mosm/kg of H_2_O (*n* = 3, *p* = 0.006) but did not change significantly after 10 min of hypotonic challenge at 140 mosm/kg of H_2_O. It is important to confirm that this response is not due to a general effect of hypotonicity on vesicular membrane proteins or changes in protein availability due to membrane unfolding associated with cellular swelling. To discount these, we measured surface expression of the astrocytic excitatory amino acid transporter EAAT1. Surface expression of EAAT1 did not change significantly with either hypotonic treatment (*n* = 3, *p* = 0.37), and there was no significant effect on cell viability measured by trypan blue exclusion (data not shown).

**FIGURE 2.**
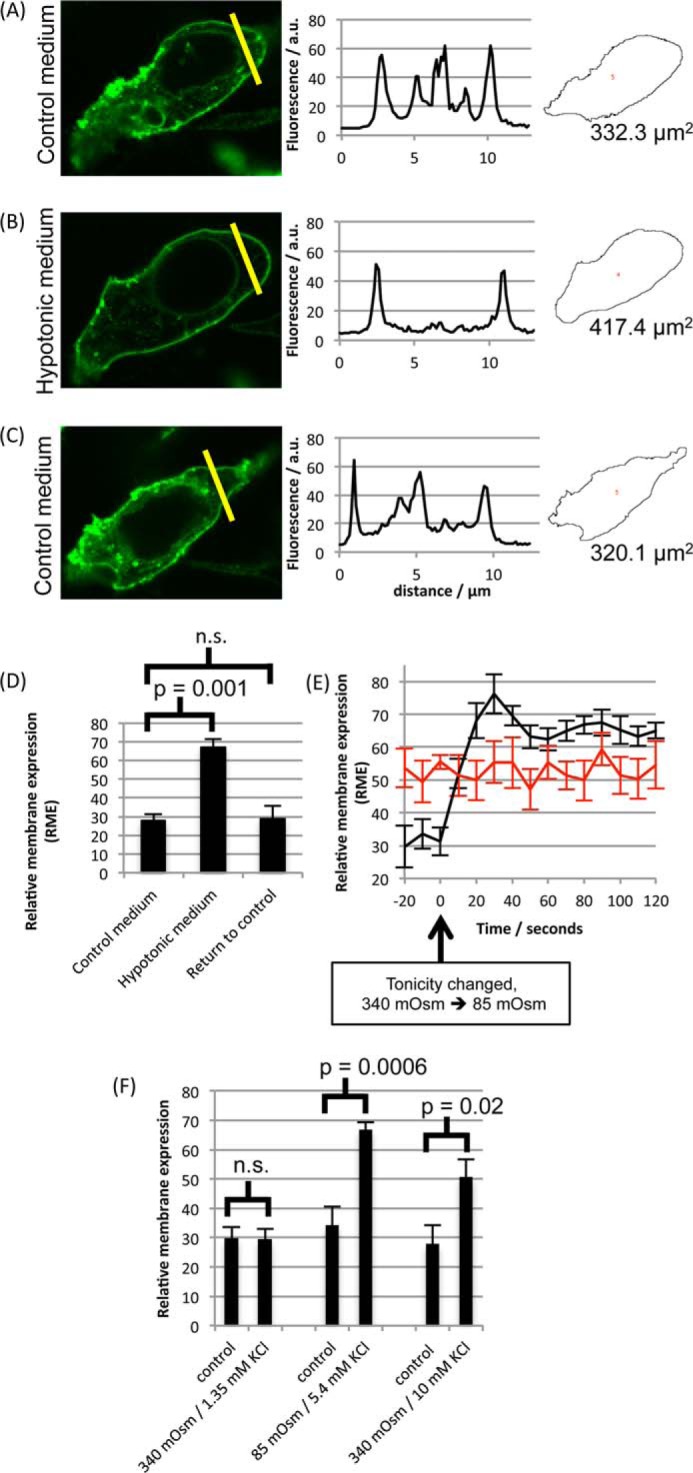
**AQP4-GFP relocalization in HEK293 cells.** *A–C*, representative fluorescence micrographs of AQP4-GFP fusion proteins in HEK293 cells following exposure to isotonic medium (340 mosm/kg of H_2_O) (*A*), a 30-s exposure to hypotonic medium (85 mosm/kg of H_2_O) (*B*), and return to isotonic extracellular environments (*C*), with fluorescence intensity profiles across the *yellow lines* and cross-sectional areas calculated using ImageJ. *a. u.*, arbitrary units. *D*, mean RME in the three conditions. Three line profiles were calculated per cell, and at least three cells per image were analyzed for each experimental repeat. *n* = 3. *p* values are from paired t tests with Bonferroni's correction following analysis of variance. *n.s.*, not significant. *E*, RME of AQP4-GFP fusion proteins in HEK293 cells (*black curve*, *n* = 3), measured by confocal fluorescence microscopy at a frame rate of 0.1 s^−1^, changed on a timescale of ∼30 s in response to reduction of extracellular tonicity from 340 mosm/kg of H_2_O to 85 mosm/kg of H_2_O, whereas membrane expression of AQP3-GFP fusion proteins did not change (*red curve*, *n* = 3). *F*, translocation is not due to a reduction in extracellular potassium. Extracellular potassium reduction (*left pair* of data points) and hypotonicity (*central pair* of data points) were applied independently by diluting media 4-fold with either isotonic NaCl (170 mm = 340 mosm) or 5.4 mm KCl in distilled H_2_O. Extracellular potassium was also increased to 10 mm in isotonic conditions (*right pair* of data points). All data are presented as mean ± S.E.

**FIGURE 3.**
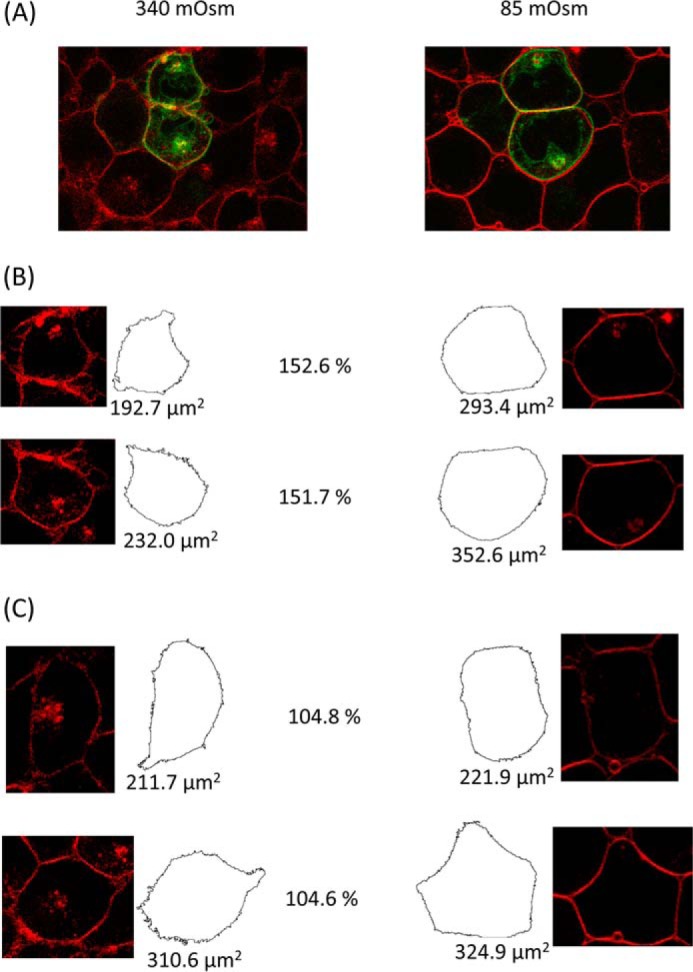
**Cell swelling of AQP4-transfected cells.** AQP4-GFP-transfected HEK293 cells were imaged using the GFP tag and FM4-64, a fluorescent membrane marker. *A*, two-color fluorescence micrograph of AQP4-GFP-transfected cells (*green*) loaded with FM4-64 (*red*), before and after reduction of extracellular osmolality from 340 mosm/kg of H_2_O to 85 mosm/kg of H_2_O. *B*, representative cross-sectional areas of transfected cells before and after hypotonic challenge, calculated using a particle detection algorithm. The post-hypotonic challenge area as a percentage of the pre-challenge area is shown between each pair of images. *C*, representative cross-sectional areas of non-transfected cells from the same image.

Primary astrocytes suffer from poor transfection efficiencies and slow growth. HEK293 cells were therefore used to further investigate AQP4 subcellular relocalization because they are much more tractable for screening cellular inhibitors and protein mutants, as demonstrated previously by our discovery of the mechanism of tonicity-induced relocalization of human AQP1 using GFP-tagged proteins (12, 13). The distribution of AQP4-GFP fusion proteins transfected into HEK293 cells was altered by changes in tonicity of the external environment. Live-cell confocal microscopy (Fig. 2) showed a rapid translocation of AQP4 to the cell surface in response to hypotonicity (following a 30-s exposure from 340 mosm/kg of H_2_O to 85 mosm/kg of H_2_O). This effect was fully reversible. Surface expression of AQP4-GFP in fluorescence micrographs was quantified by calculating the RME as described previously (13). Briefly, the difference between the average membrane fluorescence and the average intracellular fluorescence was calculated, and this was normalized to the maximum fluorescence intensity (cells with protein evenly distributed between membrane and intracellular compartments have an RME of 0, and cells with 100% of the protein at the membrane have an RME of 100). Fig. 2 shows that relative membrane expression changed from 27.86 ± 3.52 to 67.11 ± 4.47, *n* = 3, *p* = 0.001. The change in AQP4-GFP localization upon changing the extracellular tonicity from 340 mosm/kg of H_2_O to 85 mosm/kg of H_2_O happened on a timescale of 30 s. The change in RME was not due to a dilution effect or an artifact of the GFP tag as AQP3-GFP fusion proteins showed a similar distribution between membrane and cytoplasm, but no significant change in RME in response to reduced tonicity. The translocation response was not due to a reduction in extracellular potassium concentration as isotonic reduction of [K^+^]*_o_* had no effect on RME (Fig. 2*F*, *left*), whereas hypotonicity in the presence of constant [K^+^]*_o_* had the same effect as hypotonicity induced by dilution of all solutes (Fig. 2*F*, *center*). Furthermore, elevation of [K^+^]*_o_* to 10 mm in isotonic conditions increased AQP4 RME from 27.8 ± 6.3 to 50.6 ± 6.1 in agreement with published data (17) (Fig. 2*F*, *right*). AQP4-GFP-transfected cells swelled, leading to a 45 ± 1% increase of cross-sectional area after 1 min as compared with a 5 ± 1% increase for non-transfected cells in the same images (Fig. 3).

##### There Is a Threshold Tonicity for the Translocation Response

**FIGURE 4.**
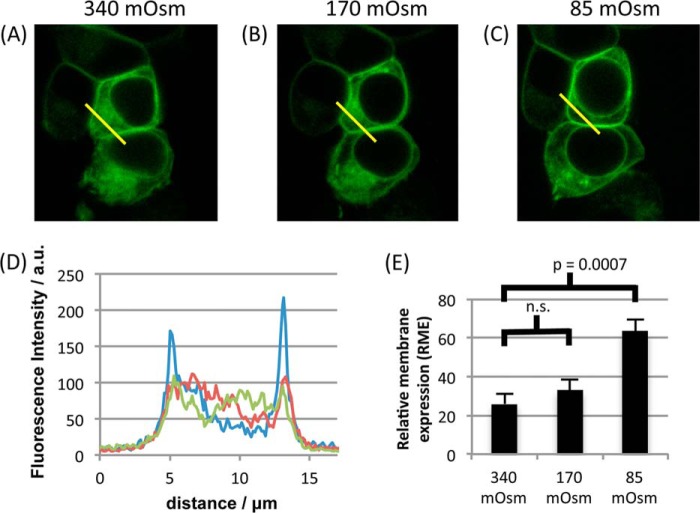
**Tonicity dependence of AQP4 relocalization.** *A–C*, AQP4-GFP-transfected HEK293 cells were imaged by confocal microscopy in a solution of 340 mosm/kg of H_2_O (*A*), 170 mosm/kg of H_2_O (*B*), and 85 mosm/kg of H_2_O (*C*). Capture of each image was started 30 s after the change in tonicity. For 170 mosm, cells were allowed to equilibrate for 10 min to check for a slower translocation response. No difference was observed between 30 s and 10 min images. *D*, representative fluorescence profiles along the *yellow lines* in *A*, *B*, and *C. a. u.*, arbitrary units. *E*, mean RME for the three tonicities, averaged over three profiles/cell and at least three cells/experimental repeat, *n* = 3. *p* values are from paired t tests with Bonferroni's correction following analysis of variance. All data are presented as mean ± S.E. *n.s.*, not significant.

AQP4 translocation in primary astrocytes was observed following a reduction of the extracellular tonicity to 85 mosm/kg of H_2_O (from 340). Reduction to 170 mosm/kg of H_2_O had no effect (Fig. 1). Fig. 4 shows that this phenomenon was reproduced in HEK293 cells. RME was 25.98 ± 5.32 in control medium, 33.07 ± 5.24 in 170 mosm hypotonic medium (not significantly different from control), and 63.61 ± 6.16 in 85 mosm hypotonic medium, *p* = 0.0007 as compared with 340 mosm, after Bonferroni's correction, *n* = 3.

##### Both AQP4 Isoforms Are Relocalized Equally

**FIGURE 5.**
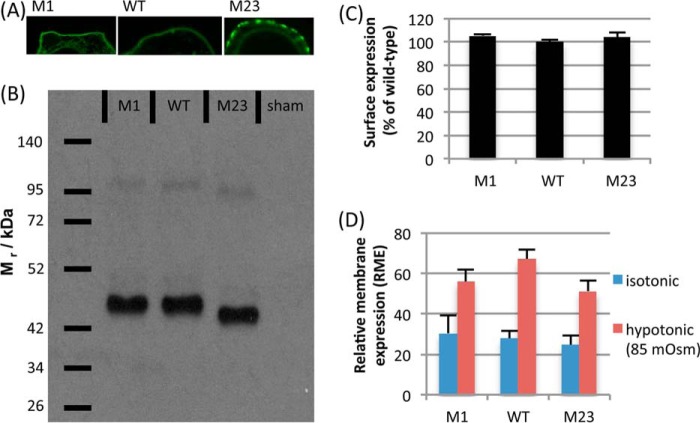
**Both AQP4 isoforms are relocalized.** Both the M1 and the M23 isoforms of AQP4 are translocated to the cell surface upon hypotonic stimulus. *A*, membrane organization of AQP4-GFP transfected into HEK293 cells. The M1 isoform has a homogeneous membrane distribution, whereas M23 clusters into orthogonal arrays of particles. *B*, SDS-PAGE of protein transcribed in HEK293 cells from the AQP4 wild-type mRNA, AQP4 M1 construct, and AQP4 M23 construct. No M23 protein was detected in the wild-type sample. *C*, there was no significant difference in constitutive surface expression between the three constructs. *D*, both isoforms of AQP4 are relocalized in response to reduction of the extracellular osmolality to 85 mosm/kg of H_2_O (M1: *p* = 0.002, *n* = 3. M23: *p* = 0.004, *n* = 3. WT: *p* = 0.001, *n* = 3.). All data are presented as mean ± S.E.

AQP4 exists in two isoforms: a long M1 form and a shorter M23 form that lacks the initial 22 amino acids of M1. Both proteins can be translated from the same full-length transcript by a “leaky scanning” mechanism, and different cell types express different M1/M23 ratios (18) via an unknown regulatory mechanism. To determine whether both isoforms or a single isoform was expressed from the wild-type AQP4 mRNA in our HEK293 system, we created constructs lacking either the M1 or the M23 translation initiation sites and compared the molecular weights of the resulting proteins with that translated from the wild-type mRNA, using SDS-PAGE. Fig. 5 shows that only the M1 isoform was present in HEK293 cells transfected with the wild-type AQP4 mRNA. M1 and M23 constructs had basal surface expression (*i.e.* at 340 mosm) equivalent to the wild type, measured using cell surface biotinylation. The confocal microscopy experiments described previously were repeated using the M1 and M23 constructs. M23 formed the punctate orthogonal arrays of particles that have been described previously for this isoform (19). RME for M23 changed from 24.58 ± 4.85 at 340 mosm/kg of H_2_O to 51.15 ± 5.25 at 85 mosm/kg of H_2_O, *n* = 3 *p* = 0.004. Although the hypotonic RME is lower than for the wild type, it is not significantly so (*p* = 0.33). This suggested that both isoforms are equally translocated to the surface in response to hypotonic stimulus.

##### Calmodulin, Calcium, and Protein Kinase A Regulate AQP4 Relocalization

AQP surface expression is known to be regulated by numerous cell signaling mechanisms. These include kinases, calcium channels, and cytoskeletal reorganization (11). AQP4 relocalization following hypotonic challenge was compared in the presence of a number of inhibitory compounds. These are described in Table 1. These data suggest that a combination of PKA kinase activity and calcium signaling mechanisms is required for AQP4 translocation. Prevention of the translocation response by inhibitors correlated with a reduction in cell swelling, suggesting a functional increase in membrane water permeability.

**TABLE 1:** **Relative membrane expression of AQP4-GFP in response to hypotonic stimulus in the presence of inhibitors** Translocation of AQP4-GFP in HEK293 cells in the presence of various inhibitors, measured by confocal microscopy and subsequent image analysis, is shown. Cells were imaged before and after reduction of the extracellular osmolality to 85 mosm/kg of H_2_O. Each RME value is an average over three independent experiments, with each experiment analyzing at least 3 cells/micrograph and 3 line profiles/cell. *p* < 0.05 was considered statistically significant (*). Abbreviations: PKCi, PKC inhibitor; PKAi, PKA inhibitor.

Inhibitor/control media	Isotonic RME (± S.E.)	Hypotonic RME (± S.E.)	Mean % of area change (± S.E.)	Translocation
Untreated control	28.77 (6.78)	78.84 (1.84)*	145.19 (1.11)*	Yes
Non-specific kinase inhibitor (hypericin)	21.85 (6.80)	38.22 (5.58)	103.50 (2.05)	No
PKC (Myr-PKCi)	27.27 (4.73)	55.28 (3.97)*	141.00 (2.05)*	Yes
PKA (Myr-PKAi)	25.62 (5.16)	33.66 (6.62)	102.97 (5.21)	No
Calcium-free media	26.69 (6.03)	32.16 (4.48)	111.60 (1.92)	No
Calmodulin inhibitor (trifluoperazine)	27.23 (7.11)	31.40 (5.91)	108.61 (5.47)	No
Calmodulin inhibitor (W7)	16.86 (8.43)	22.11 (8.14)	110.31 (14.21)	No

##### Ser-276 Phosphorylation Is Required for Relocalization

**FIGURE 6.**
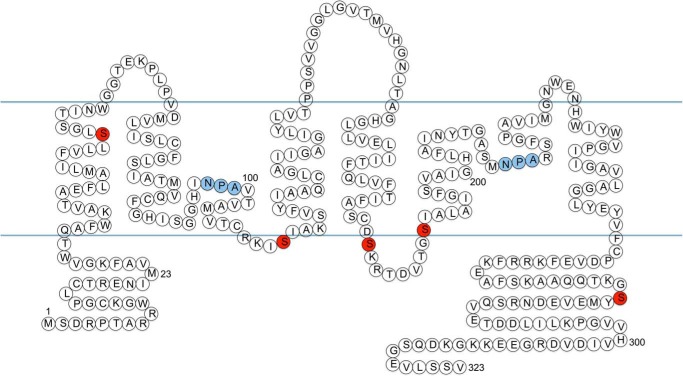
**Phosphomimetic mutagenesis of AQP4.** Locations within the AQP4 structure of identified putative PKA sites that were mutated (*red*) and the AQP family conserved NPA motifs (*blue*) are shown.

The data in Table 1 suggest a key role for PKA (but not PKC) in mediating AQP4 translocation. Using kinase site prediction software (NetPhosK 1.0 (20)), five serine residues were identified as PKA consensus sites. These were Ser-52, Ser-111, Ser-180, Ser-188, and Ser-276. The locations of these residues in the AQP4 structure are shown in Fig. 6.

The PKA consensus sites were individually mutated to alanine (to block phosphorylation) and aspartate (to act as a phosphomimetic). The effects of these mutations on the tonicity-induced relocalization of AQP4 are tabulated in Table 2. Only mutations to Ser-276 had any effect on hypotonicity-induced translocation. Substitution of Ser-276 with alanine (S276A) blocked hypotonicity-induced translocation, whereas substitution with aspartate (S276D) had no effect, suggesting that PKA phosphorylation at Ser-276 is necessary but not sufficient for translocation to occur or that S276D is a poor phosphomimetic. To determine whether this single phosphorylation event was the only mechanistic effect of PKA, translocation of the S276D mutant was measured in the presence of a myristoylated PKA inhibitory peptide (Myr-PKI). Unlike the wild-type protein, for which the translocation was inhibited by Myr-PKI, S276D still translocated to the membrane in the presence of the PKA inhibitor. This shows that S276D is a phosphomimetic and that PKA phosphorylation of AQP4 at Ser-276 is a key step in the AQP4 translocation signaling pathway. Although the S52A mutant behaved like wild-type AQP4, S52D did not translocate, but also had greatly reduced basal surface expression in comparison with wild-type AQP4 and other phosphomimetic mutants. Low surface expression prevented calculation of the RME and measurement of cross-sectional area in confocal micrographs. S52D appeared to be localized throughout the cytoplasm (rather than degraded or held as inclusion bodies), suggesting that the protein is not simply grossly misfolded. This may reflect a functional relevance, possibly interrupting processing through the Golgi apparatus or post-Golgi trafficking to the cell surface, which may be worthy of further study.

**TABLE 2:** **Relative membrane expression of AQP4 phosphorylation mutants in response to hypotonic stimulus** Translocation of phosphomimetic and phospho-blocking mutants of AQP4-GFP in HEK293 cells, measured by confocal microscopy and subsequent image analysis, is shown. Cells were imaged before and after reduction of the extracellular osmolality to 85 mosm/kg of H_2_O. Each RME value is an average over three independent experiments, with each experiment analyzing at least 3 cells/micrograph and 3 line profiles/cell. Constitutive surface expression was measured by cell surface biotinylation and normalized to wild-type expression. *p* < 0.05 was considered statistically significant (*). Abbreviation: NA, not applicable.

Mutant	Isotonic RME (± S.E.)	Hypotonic RME (± S.E.)	Mean % of area change (± S.E.)	Translocation	Constitutive surface expression (% of wild-type)
S52A	34.18 (4.06)	59.34 (5.53)*	127.03 (5.99)*	Yes	98.1 (5.3)
S52D	NA	NA	NA	No	17.0 (6.3)*
S111A	36.24 (9.95)	62.43 (5.42)*	130.69 (4.76)*	Yes	94.8 (6.4)
S111D	31.17 (4.22)	52.99 (5.41)*	120.62 (8.40)*	Yes	91.4 (4.6)
S180A	35.43 (7.78)	69.03 (6.94)*	142.65 (7.78)*	Yes	109.8 (4.5)
S180D	36.86 (6.07)	53.56 (4.96)*	148.16 (16.74)*	Yes	99.7 (5.0)
S188A	34.17 (7.21)	63.31 (7.00)*	137.01 (9.17)*	Yes	98.6 (3.2)
S188D	31.64 (9.11)	59.18 (5.60)*	135.53 (10.47)*	Yes	104.1 (4.2)
S276A	36.61 (4.64)	36.97 (5.53)	110.82 (3.85)	No	96.8 (3.2)
S276D	25.72 (5.42)	57.93 (11.01)*	134.18 (15.10)*	Yes	83.5 (5.7)*
S276D + PKA inhibitor	24.58 (4.85)	51.14 (5.25)*	127.12 (7.34)*	Yes	86.2 (5.1)*

##### PKA Activity Is Required for Endogenous AQP4 Relocalization in Primary Astrocytes

**FIGURE 7.**
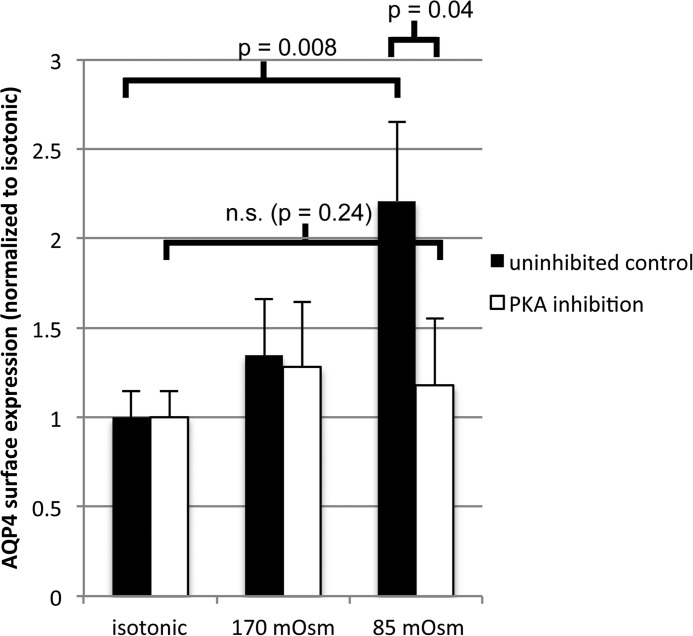
**Validation of the PKA-dependent mechanism in primary astrocytes.** Cell surface biotinylation of primary rat astrocytes exposed to hypotonic stimulus in the presence and absence of PKA inhibition and analysis of endogenous AQP4 surface expression is shown. In the absence of the inhibitor, AQP4 surface expression increased 2.2-fold between 340 and 85 mosm/kg of H_2_O, *n* = 3, *p* = 0.008. In the presence of the inhibitor, AQP4 surface expression did not change significantly between 340 and 85 mosm/kg of H_2_O, *n* = 3,*p* = 0.24. All data are presented as mean ± S.E. *n.s.*, not significant.

HEK293 cells provide a tractable model for studying the cell signaling components required for AQP4 translocation. However, it is possible that, despite sharing the translocation response, HEK293 cells and primary astrocytes translocate AQP4 by different mechanisms. To validate the mechanistic information obtained in HEK293 cells, we repeated the primary astrocyte cell surface biotinylation experiments in the presence of a PKA inhibitor. Fig. 7 shows that a 30-min pre-incubation with Myr-PKI prevented the hypotonicity-induced relocalization of endogenous AQP4 in rat primary cortical astrocytes. This suggests that the mechanistic details elucidated using HEK293 cells are physiologically relevant to the translocation of endogenous AQP4 in primary astrocytes.

## Discussion

AQP4-GFP fusion proteins in HEK293 cells and endogenous AQP4 in primary rat astrocytes rapidly relocalize to the plasma membrane in response to a reduction in local tonicity. The relocalization response takes about 30 s in HEK293 cells and is completely reversible upon return of the local tonicity to 340 mosm/kg of H_2_O. The concomitant cell swelling following AQP4 relocalization (which was reduced by inhibitors that blocked the translocation response) suggests that there is an effective increase in cell membrane water permeability. The mechanisms involved in AQP4 relocalization require PKA activity and cytoskeletal elements. Extracellular calcium is required, presumably passing through transient receptor potential (TRP) channels, which are known to mediate a calcium signaling response to osmotic cell swelling (21).

Calmodulin inhibition using two different calmodulin inhibitors (W7 and trifluoperazine) also blocked AQP4 translocation in response to hypotonicity. Calmodulin-mediated regulation of AQPs is well established. Calmodulin has been shown to inhibit AQP0 water permeability by binding directly to the C-terminal tail (22, 23), and this binding can be inhibited by AQP0 phosphorylation (24). In rat parotid cells, AQP5 was rapidly translocated to the apical membrane via acetylcholine signaling and inhibition of signaling elements downstream of calmodulin (calmodulin kinase II, myosin light chain kinase, and nitric-oxide synthase) blocked this translocation response (25). Vasopressin-induced translocation of AQP2 in collecting duct cells can be attenuated by extracellular calcium in a calmodulin-dependent manner (26). Calmodulin has also been shown to bind directly to the N-terminal tail of AQP6 (27), although the functional relevance of this interaction is unknown. We showed previously that inhibition of calmodulin prevented hypotonicity-induced relocalization of AQP1 in HEK293 cells (12). It is not clear from our data whether calmodulin binds directly to AQP4 or activates a third party protein to mediate AQP4 translocation, although a calmodulin binding site prediction tool (Calmodulin Target Database (28)) does predict that the most likely site for direct binding of calmodulin to AQP4 is the 20-residue peptide at positions 277–296, directly upstream of the Ser-276 phosphorylation site we identified.

Kinase-dependent translocation of AQPs to the plasma membrane from intracellular vesicles is an established regulatory mechanism that has been demonstrated for AQP2 in response to vasopressin signaling via PKA (29), AQP1 in response to hypotonicity via PKC (13), and AQP5 in response to acetylcholine via PKG (25). Treatment with the broad-range kinase inhibitor, hypericin, suggested a similar mechanism for AQP4 relocalization. Using specific inhibitors identified PKA activity as a requirement for tonicity-mediated translocation of AQP4. There is some evidence that PKA reduced surface availability of AQP4 in human gastric cells (14). In our systems, PKA seems to be doing the opposite.

All potential PKA sites of AQP4 were mutated, suggesting a key requirement for serine at position 276 (Ser-276) in the C-terminal tail of AQP4. Phosphorylation at this residue has been detected *in vivo* in murine AQP4 (30) and rat AQP4 (31). It was recently reported that phosphomimetic (S276D) or phospho-blocking (S276A) mutations at this residue have no effect on surface expression or water permeability, leaving the purpose of this phosphorylated residue unclear (32). Our data provide evidence of a functional role for Ser-276 phosphorylation. It has also been reported that phosphorylation at Ser-276 can increase lysosomal targeting of AQP4 upon internalization (33). This may explain the slight reduction in constitutive surface expression (to 83.5 ± 5.7%) that we measured for the S276D mutant. PKC phosphorylation of Ser-180 in response to AVP has been reported to cause AQP4 internalization (34); we did not observe any change in constitutive surface expression or translocation response for the S180D phosphomimetic mutant in our system. This may reflect the lack of an element of this internalization pathway in HEK293 cells or a species-specific effect (these experiments were done in *Xenopus* oocytes). Phosphorylation of AQP4 at Ser-111 in response to elevated extracellular potassium has been reported to increase membrane water permeability in an astrocytic cell line (17). S111A and S111D mutations had no effect in our system, and furthermore S276D translocated in the presence of a PKA inhibitor, suggesting that the only role of PKA in our system is to phosphorylate Ser-276. Although an isotonic *increase* in extracellular potassium induced AQP4 translocation in our system, which agrees with a previous study (17), the hypotonicity-induced translocation of AQP4 was independent of any reduction in extracellular potassium associated with the hypotonic challenge. Although it is probable that Ser-276 is phosphorylated in our primary astrocyte experiments, we cannot rule out a difference in phosphorylation sites between different cells or a synergistic effect of several kinase sites in the primary cells.

**FIGURE 8.**
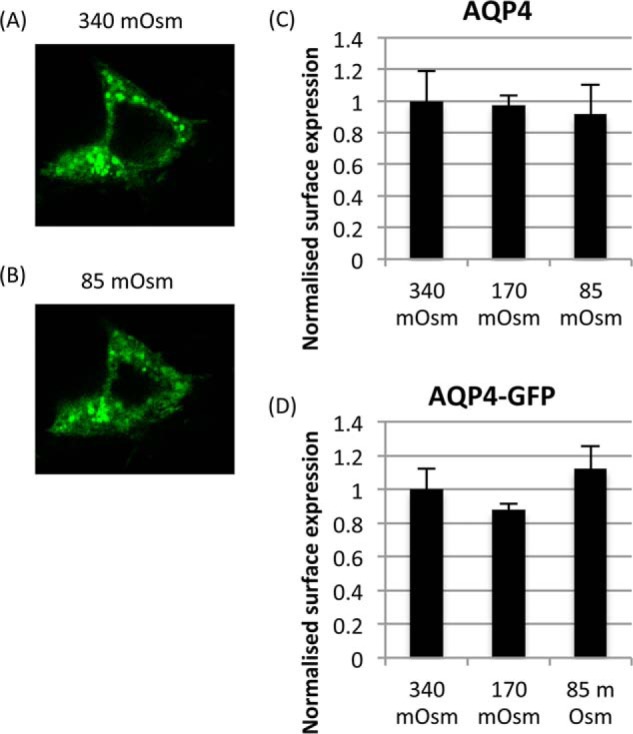
**Lack of AQP4 translocation response in the U373 glioblastoma cell-line.** *A* and *B*, representative confocal micrographs of an AQP4-GFP-transfected U373 cell exposed to isotonic (340 mosm/kg of H_2_O) media (*A*) and 5-min exposure to hypotonic (85 mosm/kg of H_2_O) media (*B*). *C* and *D*, cell surface biotinylation of U373 cells transfected with AQP4 (*C*) and AQP4-GFP (*D*). No statistically significant change was observed between any of the treatment groups (340, 170, and 85 mosm/kg of H_2_O), *n* = 3. Data are presented as mean ± S.E.

Interestingly, we found that both endogenous and transfected AQP4 (with and without GFP) did not relocalize in response to any tonicity change in the U373 glioblastoma cell line (Fig. 8). There is some evidence that a dominant-negative splice variant of AQP4 lacking exon 4, which codes for the half-helix containing the second NPA motif, can inhibit surface expression of either the M1 or the M23 isoforms of AQP4 when co-expressed (35). This was reported in muscle cells, but it may be possible that dysregulation of this isoform inhibits surface expression in U373 cells. People with glioblastoma have a very poor prognosis. The cells are very invasive, and edema is a key problem. This may be worthy of further investigation to determine whether this is a peculiarity of the U373 cell line or representative of glioblastoma physiology.

To conclude, the data reported here provide a mechanistic explanation for our discovery of the physiological relocalization of AQP4 in response to changes in local tonicity. A change in local tonicity is the key driver of cell swelling in stroke and contributes to the effects of cytotoxic edema following traumatic brain injury. Modulating the surface expression of AQP4 rather than trying to directly block its pore is a novel platform for developing therapies for these devastating conditions.

## Footnotes

4 The abbreviations used are:

AQP: aquaporin
Myr: myristoylated
PKI: protein kinase inhibitor
RME: relative membrane expression
AVP: Arg-8 vasopressin
ER: endoplasmic reticulum.
